# Development of a Machine Learning-Based Prediction Model for Chemotherapy-Induced Myelosuppression in Children with Wilms’ Tumor

**DOI:** 10.3390/cancers15041078

**Published:** 2023-02-08

**Authors:** Mujie Li, Quan Wang, Peng Lu, Deying Zhang, Yi Hua, Feng Liu, Xing Liu, Tao Lin, Guanghui Wei, Dawei He

**Affiliations:** 1 Department of Urology, Children’s Hospital of Chongqing Medical University, Chongqing 400015, China; 2 Chongqing Key Laboratory of Children Urogenital Development and Tissue Engineering, Chongqing Key Laboratory of Pediatrics, Ministry of Education Key Laboratory of Child Development and Disorders, National Clinical Research Center for Child Health and Disorders, International Science and Technology Cooperation Base of Child Development and Critical Disorders, Chongqing 400014, China; 3 Department of Cardiothoracic Surgery, Children’s Hospital of Chongqing Medical University, Chongqing 400015, China; 4 Chongqing Higher Institution Engineering Research Center of Children’s Medical Big Data Intelligent Application, Chongqing 401331, China

**Keywords:** chemotherapy-induced myelosuppression, Wilms’ tumor, artificial intelligence, machine learning, prediction model

## Abstract

**Simple Summary:**

Wilms’ tumor is the most common renal malignant tumor in children, and chemotherapy is an indispensable part of the treatment for most Wilms’ tumor patients. Chemotherapy-induced myelosuppression is the most common and serious toxicity of chemotherapy, which can hinder the process of chemotherapy and even endanger life. However, there is a lack of tools to predict chemotherapy-induced myelosuppression. We herein develop a model based on machine learning that can effectively predict the risk of chemotherapy-induced myelosuppression in children with Wilms’ tumor, offering the possibility to identify children with high risk of chemotherapy-induced myelosuppression early and take preventive strategies.

**Abstract:**

**Purpose:** Develop and validate an accessible prediction model using machine learning (ML) to predict the risk of chemotherapy-induced myelosuppression (CIM) in children with Wilms’ tumor (WT) before chemotherapy is administered, enabling early preventive management. **Methods:** A total of 1433 chemotherapy cycles in 437 children with WT who received chemotherapy in our hospital from January 2009 to March 2022 were retrospectively analyzed. Demographic data, clinicopathological characteristics, hematology and blood biochemistry baseline results, and medication information were collected. Six ML algorithms were used to construct prediction models, and the predictive efficacy of these models was evaluated to select the best model to predict the risk of grade ≥ 2 CIM in children with WT. A series of methods, such as the area under the receiver operating characteristic curve (AUROC), the calibration curve, and the decision curve analysis (DCA) were used to test the model’s accuracy, discrimination, and clinical practicability. **Results:** Grade ≥ 2 CIM occurred in 58.5% (839/1433) of chemotherapy cycles. Based on the results of the training and validation cohorts, we finally identified that the extreme gradient boosting (XGB) model has the best predictive efficiency and stability, with an AUROC of up to 0.981 in the training set and up to 0.896 in the test set. In addition, the calibration curve and the DCA showed that the XGB model had the best discrimination and clinical practicability. The variables were ranked according to the feature importance, and the five variables contributing the most to the model were hemoglobin (Hgb), white blood cell count (WBC), alkaline phosphatase, coadministration of highly toxic chemotherapy drugs, and albumin. **Conclusions:** The incidence of grade ≥ 2 CIM was not low in children with WT, which needs attention. The XGB model was developed to predict the risk of grade ≥ 2 CIM in children with WT for the first time. The model has good predictive performance and stability and has the potential to be translated into clinical applications. Based on this modeling and application approach, the extension of CIM prediction models to other pediatric malignancies could be expected.

## 1. Introduction

Wilms’ tumor (WT) is the most common renal malignancy in children and has the second highest incidence of pediatric primary abdominal malignancies. Although multidisciplinary treatments have advanced, recurrence occurs in approximately 15% of children with WT, and the survival rate after recurrence is only about 50% [1,2,3]. As the surgical resection of pediatric tumors is often difficult, chemotherapy is an indispensable part of the treatment for most WT patients.

However, chemotherapy drugs have many toxicities and side effects. Chemotherapy-induced myelosuppression (CIM) is the most common and severe toxicity of chemotherapy for tumors, typically manifesting as anemia, neutropenia, thrombocytopenia, and/or lymphopenia [4,5,6,7], leading to an increased risk of life-threatening infection, fatigue, and potential bleeding [8,9]. CIM often forces children to interrupt or postpone their chemotherapy course, severely compromising the effectiveness of treatment and even leading to death due to CIM-related complications. Studies have reported that the mortality rate related to grade 4 CIM can reach 4–12% [10]. Therefore, early identification of children at high risk of CIM and timely implementation of corresponding preventive and therapeutic measures can not only improve the effectiveness of tumor treatment, but also significantly reduce the disease burden caused by the related complications [11].

Studies have shown that risk factors for CIM include age, nutritional status, poor liver and kidney function, low baseline white blood cell count (WBC), chemotherapy cycles, etc. [12,13,14,15]. Various mathematical models for predicting CIM or febrile neutropenia (FN) have been proposed [16,17,18] and successfully applied to predict dynamic changes in neutrophil count [19,20]. However, these studies focused on predicting the risk of FN in adult tumors such as breast cancer, small cell lung cancer, and colorectal cancer [14,21,22].

The predictors of CIM in pediatric malignant solid tumors, especially in WT, have not been reported. In addition, most of the pharmacokinetic mathematical models developed in these studies focus on predicting CIM/FN caused by a single drug, making it difficult to extend to pediatric tumors requiring multidrug combination therapy. Moreover, the application of these models requires repeated and frequent monitoring of changes in hematological parameters and drug concentrations, such invasive tests are often unacceptable to children and parents [20,22], and the relatively backward economic and medical levels in developing countries seem to make the implementation of such monitoring strategies more difficult.

Therefore, CIM or FN prediction models reported in the existing studies are difficult to widely apply to predict CIM in children with WT. It is necessary to develop a CIM prediction model for children with WT that is easy to use and has good prediction efficiency.

At present, artificial intelligence (AI) has been widely applied in the medical field. Machine learning (ML), as a branch of AI, can overcome the shortcomings of traditional logistic regression and mathematical models, and has a strong ability for feature recognition, classification, and prediction [23]. The models established based on machine learning have been successfully used in predicting the prognosis of various tumors or diseases, which presented good predictive ability [24,25,26]. Shibahara et al. collected pretreatment clinical data of glioma patients treated with nimustine hydrochloride (ACNU), and further successfully established a prediction model of CIM using machine learning, as well as describing the relationship between myelosuppression and hematopoietic stem cells (HSCs) [27]. In our study, various premedication clinical data in each chemotherapy cycle of WT children with a large sample size from the clinical big data platform of our hospital were collected, including blood cells baseline level, liver and kidney function indicators, tumor stage, body weight, body surface area and other variables, and six ML algorithms were used to construct CIM prediction models. Meanwhile, further evaluation of each model was carried out to select the model with the best prediction performance, which can help doctors identify children with WT at high risk of CIM early and develop individualized strategies for prevention, treatment, and follow-up to reduce the disease burden and improve prognosis.

## 2. Methods

### 2.1. Patients

The data of patients with WT who received chemotherapy in our hospital from January 2009 to March 2022 were collected from our hospital’s clinical big data platform. Inclusion criteria: (1) younger than 18 years old; (2) patients diagnosed with WT; (3) patients having received at least one cycle of chemotherapy; (4) patients having received at least one routine blood test and biochemical blood test before and after chemotherapy. Exclusion criteria: (1) patients with other hematologic diseases or a history of HIV infection or stem cell transplantation; (2) patients with incomplete medical records (missing more than 50% of variables used for analysis); (3) patients with treatment interruption. 

### 2.2. Collection and Definition of Variables

#### 2.2.1. General Variables

Variables such as demographic data, clinicopathological characteristics, the laboratory examination, and medication information after each admission were collected as follows: age, gender, height, weight, tumor stage, COG grade, the routine hematologic index and biochemical index, routine urinalysis, the type of chemotherapy drugs used, chemotherapy cycles, etc.

#### 2.2.2. Outcome Indicators

The occurrence of grade ≥ 2 CIM was taken as the outcome indicator. According to the National Cancer Institute Common Terminology Criteria for Adverse Events (CTCAE) version 5.0, if one of the following 4 criteria is met after chemotherapy, it can be defined as grade ≥ 2 CIM: (1) WBC < 3.0 × 10^9^/L; (2) absolute neutrophil count (ANC) < 1.5 × 10^9^/L; (3) hemoglobin level (Hgb) < 100 g/L; (4) platelet count (PLT) < 75 × 10^9^/L.

#### 2.2.3. Calculation of Composite Variables

(1) Systemic immune-inflammation index (SII) = PLT × ANC/absolute lymphocyte count (ALC) [28]
(2) Neutrophil to lymphocyte ratio (NLR) = ANC/ALC
(3) Platelet to lymphocyte ratio (PLR) = PLT/ALC
(4) Body surface area (BSA) = 0.035 × body weight + 0.1 (body weight ≤ 30 kg)

BSA = 1.05 + (body weight − 30) × 0.02 (body weight > 30 kg)

#### 2.2.4. Derived Variables

Coadministration of highly toxic chemotherapy drugs refers to any high hematologic toxicity chemotherapy drugs used during that chemotherapy cycle.

Chemotherapy drugs are divided into two categories according to the level of hematological toxicity [29,30]: (1) high: cyclophosphamide (CTX), ifosfamide, doxorubicin, epirubicin, actinomycin D, carboplatin, etoposide, topotecan, vindesine; (2) moderate/low: cisplatin, vincristine, bleomycin, fluorouracil.

### 2.3. Data Preprocessing

#### 2.3.1. Quality Control of Samples

Each chemotherapy cycle of each WT patient was taken as a separate sample. The missing rate of each sample characteristic variable was counted, and 50% was selected as the threshold value according to the distribution of each sample characteristic variable and modeling requirements. If 50% or more of all characteristic variables were missing simultaneously, the sample characteristic variable was considered seriously missing and met the exclusion criteria.

#### 2.3.2. Imputation Methods of Missing Data

For clinical characteristic variables, after the sample size was determined, the missing rate of each characteristic variable was checked, and 20% was selected as the threshold according to the modeling requirements. If the missing rate of the characteristic variable exceeds 20%, the variable will be deleted and not included in the model construction. Other missing categorical variables were imputed with the mode while missing continuous variables were imputed with the median. In addition, chemotherapy drugs with a relative frequency of medication less than 5% were also deleted and not included in the model construction (relative frequency of medication = frequency of drug use/total sample size).

### 2.4. Model Building

#### 2.4.1. Datasets and Algorithms

Extreme gradient boosting (XGB), logistic regression (LR), random forest (RF), least absolute shrinkage and selection operator (LASSO), support vector machine (SVM), and CatBoost were used to establish the ML model. R version 4.2.0 and Python version 3.7 were used for model construction and statistical analysis. Stratification was performed according to the outcome, and the data set was randomly divided into the training set and the test set at a 7:3 ratio.

#### 2.4.2. Original Variables and Variable Selection

Information value (IV) was used as a correlation indicator, which can be used to measure the difference in the distribution of a variable between the two groups of samples to characterize the predictive ability of the variable on the outcome [31]. The threshold value of IV was set as 0.2, and variables with IV less than 0.2 were deleted. Since the chemotherapy cycle and the type of chemotherapy drugs have been confirmed to be related to the occurrence of CIM, these two variables were included in the model even though their IV were less than 0.2.

For the selected variables related to the outcome, the absolute value of the correlation coefficient was calculated to examine the collinearity, and the threshold was set as 0.8. The variable with the smaller IV was also deleted from the collinear variables exceeding the threshold.

#### 2.4.3. Modelling Procedure

Fivefold cross-validation (CV) was used to divide the CV training set and the CV validation set inside the training set, then the optimal hyperparameter of the model was obtained using Bayesian optimization. According to the optimal hyperparameter, the model was trained again on the entire training set to obtain the final model, and further evaluated the models’ prediction performance on the training set and test set.

The area under the curve (AUC), sensitivity (TPR), specificity (TNR), precision (ACC), and precision (PPV) of the receiver operating characteristic curve (ROC) were used to characterize the fitting and accuracy of the model. Population stability index (PSI) was used to measure the stability of the model in the training set and validation set [32]. (PSI < 0.1, the model is stable; PSI: 0.1~0.25, the model is slightly unstable; PSI > 0.25, the model is unstable). Hosmer–Lemeshow test was used to assess the calibration of models. The decision curve analysis (DCA) was used to evaluate the clinical utility of these models. Moreover, Coefficients of weight importance in the final model were provided to rank the feature importance.

#### 2.4.4. Clinical Application of the Model

In order to realize the translation of research results into clinical practice, the model was presented and applied in our hospital information system (HIS) in the form of clinical decision support system (CDSS). After the first hematological examination for each patient, the doctor preliminarily confirms the medication regimen, at which point the system backstage automatically extracts the relevant data from the HIS into the model, then calculate the risk value and present it in the CDSS. “Risk Scoring” is one of the essential modules. A patient’s risk score was calculated based on the final model score × 100, where low–medium risk was classified according to negative predictive value (NPV) = 0.8 and medium–high risk was classified according to positive predictive value (PPV) = 0.9. That is, the cutoff value for low–medium risk should ensure a negative prediction rate of >80% for low-risk patients, and the cutoff value for medium–high risk should ensure a positive prediction rate of >90% for high-risk patients.

To further improve the intuitiveness, accessibility, and practicability of the model, a brief description and the scoring basis of the model were presented in the CDSS, and the “Historical Trend” module was added to show the occurrence of CIM in previous admissions. In addition, the system can provide recommendations for possible prevention or intervention strategies based on the model scores.

### 2.5. Statistical Analysis Methods

Continuous variables were described in the form of the median (lower and upper quantile), and categorical variables were described in the form of frequency and percentage. Wilcoxon rank sum test and chi-square test were used to compare the differences between groups for continuous variables and categorical variables, respectively. *p* < 0.05 was considered statistically different.

**Figure 1:**
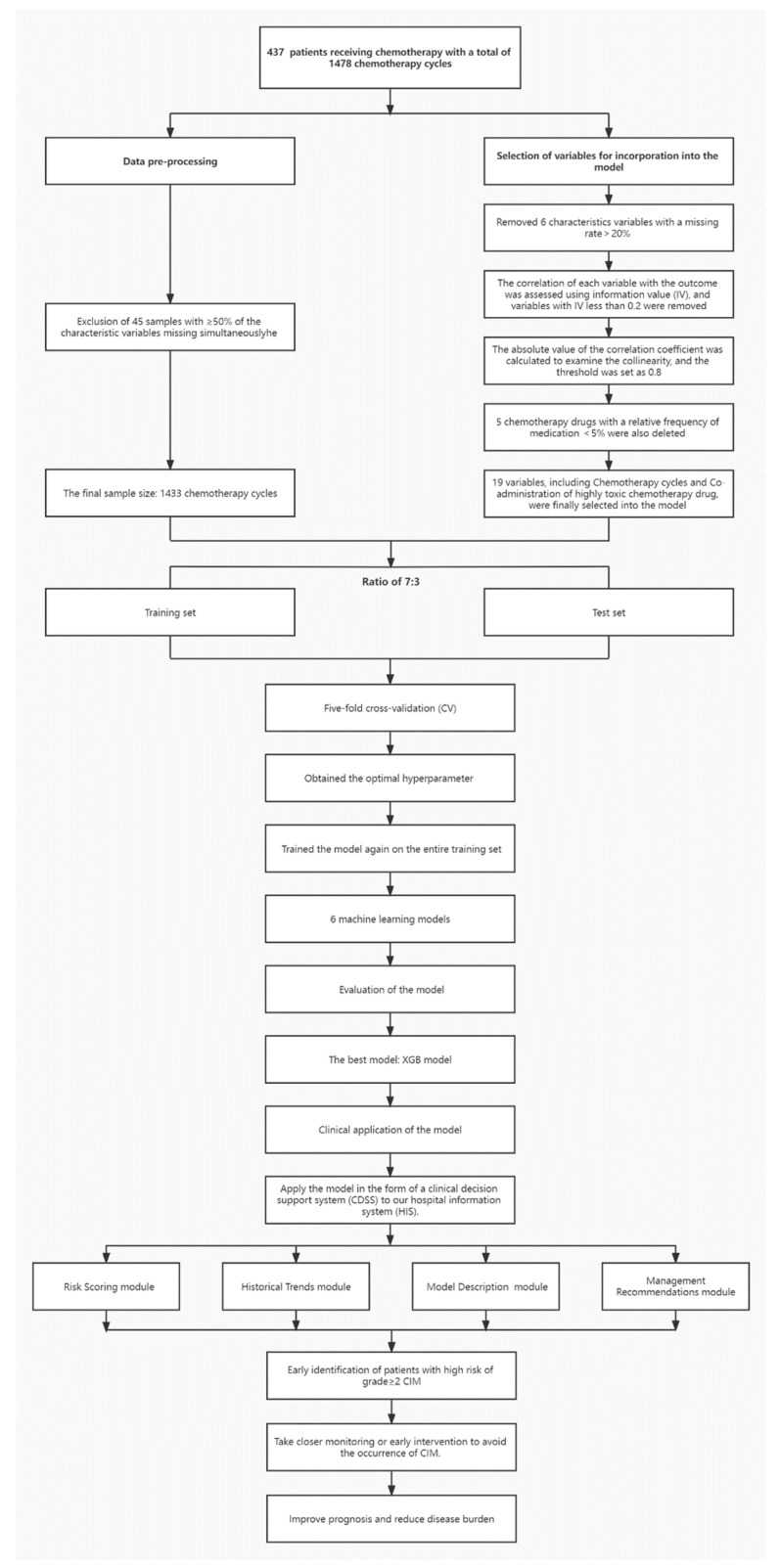
The entire modeling procedure.

The entire modeling procedure is shown in Figure 1.

## 3. Result

### 3.1. Description of Baseline Characteristics

On our hospital’s clinical big data platform, 437 cases of WT patients receiving chemotherapy were retrieved, with a total of 1478 chemotherapy cycles. According to the inclusion and exclusion criteria, 45 samples were excluded, resulting in a final sample size of 1433. According to the National Cancer Institute Common Terminology Criteria for Ad-verse Events (CTCAE) version 5.0, grade ≥ 2 CIM can be defined if one of the following four criteria is met after chemotherapy: (1) WBC < 3.0 × 10^9^/L; (2) absolute neutrophil count (ANC) < 1.5 × 10^9^/L; (3) hemoglobin level (Hgb) < 100 g/L; (4) platelet count (PLT) < 75 × 10^9^/L. The baseline characteristics of all patients and the comparison of baseline characteristics of patients in different datasets are shown in Table 1, and the comparison of baseline characteristics of patients with and without grade ≥ 2 CIM is shown in Table 2.

### 3.2. Selection of Variables during Modeling

Matching the patient’s first laboratory examination index after admission, a total of 46 clinically relevant characteristic variables were extracted, of which six characteristic variables (absolute value of basophils, percentage of basophils, cholinesterase, prealbumin, bile acids, and urine pH) had a missing rate of more than 20% and were excluded. Finally, 40 clinical characteristic variables were incorporated into the model for further screening, as shown in Table 3.

### 3.3. Selection of Chemotherapy Drugs

The relative frequency of the use of each chemotherapy drug is shown in Table 4, among which bleomycin, fluorouracil, topotecan, vindesine, and ifosfamide were excluded because the relative frequency of use was less than 5% and significantly different from that of other drugs. Thus, a total of nine variables including cisplatin, doxorubicin, epirubicin, carboplatin, etoposide, actinomycin D, cyclophosphamide, and vincristine, as well as the coadministration of highly toxic chemotherapy drugs, were incorporated into the final model.

### 3.4. Variables Finally Selected for the Model

**Figure 2:**
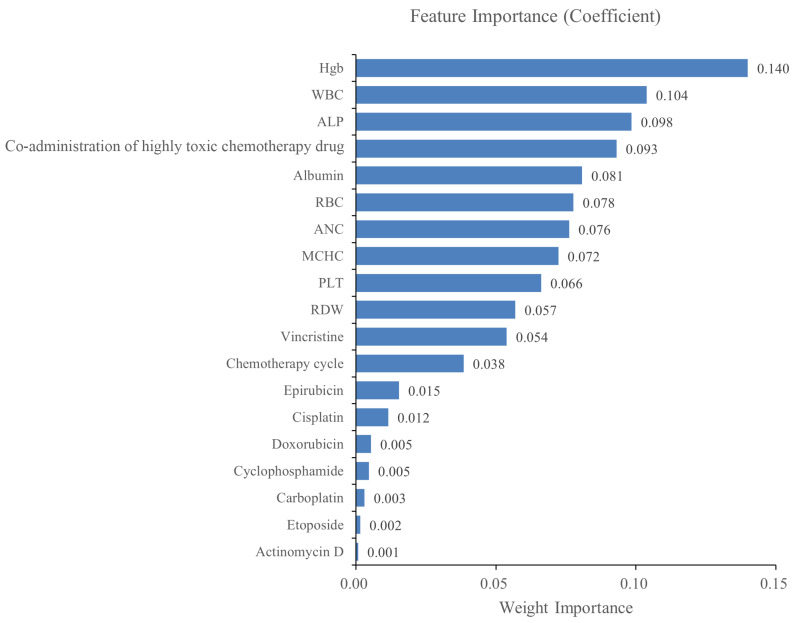
The ranking of feature importance in the XGB model. Briefly, the importance weight of a feature is the sum of the number of its occurrences in all decision trees. In other words, the more a feature is used to build a decision tree in the model, the higher its importance weight will be. Hgb: hemoglobin; WBC: white blood cell count; ALP: alkaline phosphatase; RBC: red blood cell count; MCHC: mean corpuscular hemoglobin concentration; PLT: platelet count; RDW: red blood cell distribution width.

According to the selection criteria of predictive variables, 19 variables finally incorporated into the model are shown in Table 5. In order to improve the interpretability of the final model (XGB), we ranked the feature importance of the incorporated variables. The five variables contributing the most to the model were hemoglobin (Hgb), white blood cell count (WBC), alkaline phosphatase, coadministration of highly toxic chemotherapy drugs, and albumin, as shown in Figure 2.

### 3.5. Evaluation of the Model

**Figure 3:**
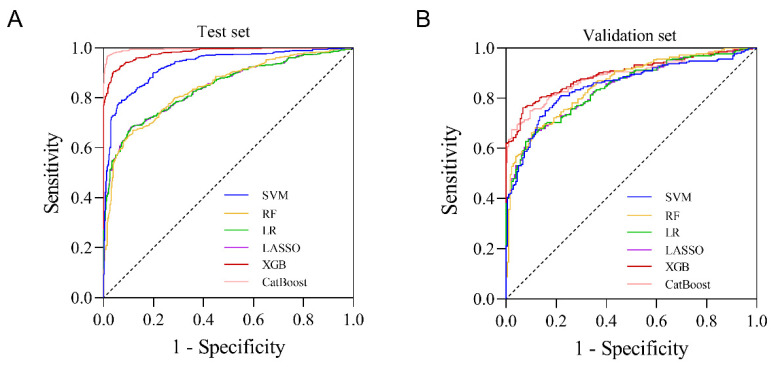
ROC curve of six ML models for predicting grade ≥ 2 CIM. (**A**) In the test set; (**B**) in the validation set. SVM: support vector machine; RF: random forest; LR: logistic regression; LASSO: least absolute shrinkage and selection operator; XGB: extreme gradient boosting.

**Figure 4:**
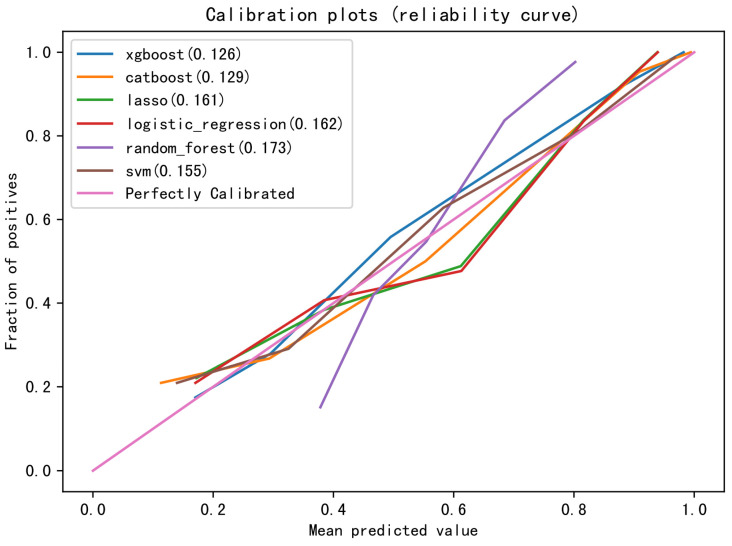
Calibration curves of the six ML models.

**Figure 5:**
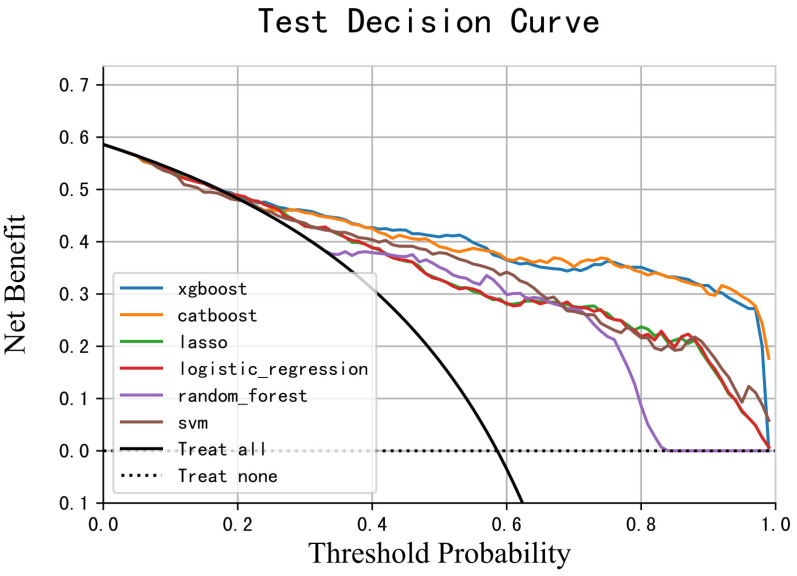
Decision curve analysis of the six ML models.

The fitting effect and authenticity evaluation results of each model are shown in Figure 3, Table 6 and Table 7, respectively. The results show that the XGB model has the best fitting effect, the largest AUC (training set: 0.981, test set: 0.896), good sensitivity (76.2%), and specificity (93.2%), and better stability. In the XGB model, the feature importance of each variable is shown in Figure 2. The five variables that contribute the most to the model are Hgb, WBC, alkaline phosphatase, coadministration of highly toxic chemotherapy drugs, and albumin. In addition, the XGB model showed the best calibration in the comparison of calibration curves of other models (Figure 4). DCA showed that the XGB model can contribute to clinical decision-making (Figure 5).

### 3.6. Clinical Application of the Model

**Figure 6:**
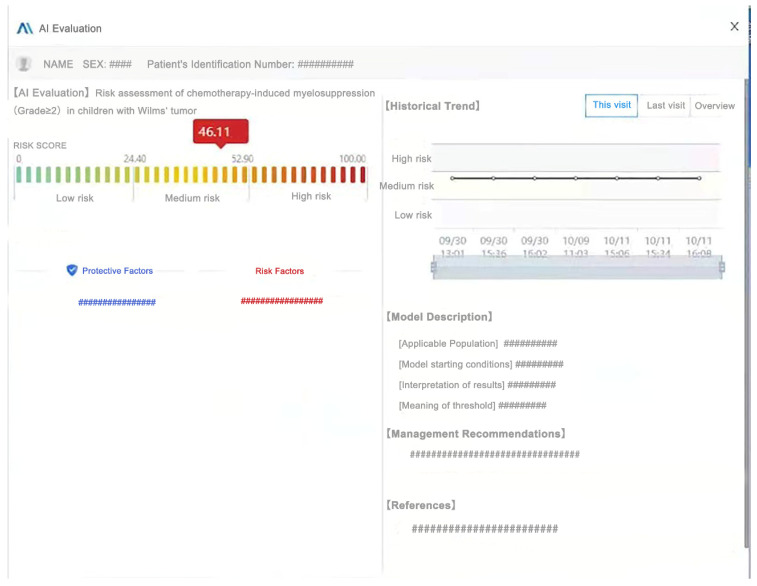
**The interface of the CIM prediction model in the form of CDSS applied in our hospital HIS. AI Evaluation:** the “AI Evaluation” module shows the risk scores of patients with grade ≥ 2 CIM calculated by the model, with the corresponding “protective factors” and “risk factors” listed below. **Historical Trend:** the “Historical Trends” module records the occurrence of CIM in previous chemotherapy cycles. **Model Description:** this module provides a detailed description of the applicable conditions and the model results. **Management Recommendations:** according to the prediction results of the model, the management suggestions automatically output by the system backstage are displayed in this module. **References:** this module presents some references.

Through a series of evaluations of the model, the XGB model with the best predictive efficacy was selected, presented, and applied in our hospital’s HIS in the form of CDSS. It includes modules such as the risk scoring and scoring basis of grade ≥ 2 CIM, model description, historical trend of the previous occurrence of CIM, and management recommendations (Figure 6). The predictive model is currently running smoothly in the HIS. Moreover, to better demonstrate how our model works in reality and to further elaborate on the clinical applicability of the model, we ran the model in our hospital HIS to assess the risk of CIM in a particular child (Supplementary Materials).

## 4. Discussion

### 4.1. CIM Is Not Rare during the Treatment of Children with WT

Chemotherapy is one of the important means of treating tumors. Currently, most chemotherapy drugs exert their effects through cytotoxicity. Cells with strong proliferative activity may be more sensitive to chemotherapy drugs, making drugs more likely to damage hematopoietic stem cells or blood cell precursors, leading to severe CIM [27,33]. A clinical consensus is that grade ≥ 2 CIM requires close monitoring and even timely intervention. Identifying patients with a high risk of grade ≥ 2 CIM before administration of chemotherapy drugs can guide doctors to timely administer granulocyte colony-stimulating factor (G-CSF) and other drugs to prevent the occurrence of CIM during the process of closely monitoring the changes in blood cells levels, which avoids the interruption of the chemotherapy course and even the occurrence of more serious complications caused by CIM [34,35]. It is also why we choose the occurrence of grade ≥ 2 CIM as the outcome indicator. In this study, grade ≥ 2 CIM occurred in 58.5% (839/1433) chemotherapy cycles. Although Castagnola et al. reported that the incidence of FN in children with central nervous system tumors was 27% [36], the outcome of the study was FN rather than CIM, and the different types of tumors studied may also affect the incidence of FN, so our findings cannot be compared with their study. Other studies have reported that the incidence of FN in solid tumors is 13–21%, while FN in hematologic tumors is about 33% [37,38,39]. Whereas most of the outcome indicators in these studies were FN, and the subjects were adults, which could not be compared with the incidence of CIM in our study. However, this also emphasizes that the incidence of CIM in children with solid tumors is still unknown and more studies are needed to fill in the gaps. In addition, more than half of the chemotherapy cycles in our study presented grade ≥ 2 CIM, which fully demonstrates that CIM is not rare in treating pediatric tumors, especially WT, and the development of early prediction models for CIM in children with solid tumors is indeed necessary.

### 4.2. Contribution of Variables to Model Prediction Results

According to the ranking of IV, 19 variables were finally included in the model. Studies have shown that chemotherapy cycles and regimens can affect the occurrence of CIM, so even if the IV of those relevant variables were less than 0.2, they were still included in our model. Feature importance is an indicator to measure the contribution of each variable to the model’s predictive result (Figure 1). In the XGB model, the Hgb level ranked first in the feature importance ranking. This seems to differ from what most studies have reported. More than one study reported that baseline WBC and ANC levels, but not Hgb levels, were the most critical risk factors for CIM or FN [14,40,41]. On the contrary, it has also been reported that a low baseline level of Hgb was associated with CIM in elderly tumor patients [42]. It has been reported that in addition to Hgb, the decrease of alkaline phosphatase, red blood cell count (RBC), and average hemoglobin concentration and the increase of red blood cell distribution width (RDW) can also reflect anemia or hematopoietic abnormalities to some extent [27,33]. Herein, except for RDW, the above five indicators were lower in the CIM group than in the without-CIM group. This may be because most of the children in this study underwent surgery before chemotherapy, and inevitable intraoperative bleeding and the consumption of the tumor on the body led to a lower baseline Hgb or RBC level before chemotherapy. While stimulated by blood loss, the proliferation of bone marrow hematopoietic cells may be more active, thus more likely to be attacked by chemotherapy drugs.

Although Aagaard et al. did not find that low levels of WBC and ANC were associated with the development of bone marrow suppression in their study [43], most studies have shown that low baseline WBC and ANC levels are risk factors for myelosuppression [12,13,14], and our findings are consistent with them: the low baseline level of WBC and ANC in the XGB model strongly predicts CIM. Due to the short cycle life of granulocytes, it is difficult for haemopoietic stem cells or haemopoietic microenvironment damaged by chemotherapy drugs to generate new granulocytes to replace the consumed granulocytes [27,30]. Hence, a low ANC level is often the earliest manifestation of CIM. Lower baseline WBC or ANC levels mean lower granulocyte reserves, meaning CIM is more likely to occur.

In addition, the low baseline level of albumin may be related to the nutritional status of patients, thus affecting the occurrence of CIM, which is also consistent with the result of another study [44].

Moreover, different patients have different chemotherapy regimens [45,46,47], and different chemotherapy regimens incorporate chemotherapy drugs with different degrees of hematological toxicity [48,49], so treating each chemotherapy regimen as a variable is unrealistic. As a result, we added the variable “Coadministration of highly toxic chemotherapy drugs” to investigate the effect of highly toxic chemotherapy drugs on the risk of developing CIM. Although its IV was small, its feature importance ranked fourth in the XGB model. It validates that chemotherapy drugs with high hematotoxicity are indeed more likely to cause CIM. Unexpectedly, the ranking of feature importance of chemotherapy drugs in the model seems to be different from our understanding of hematological toxicity of chemotherapy drugs. Low hematologic toxicity drugs such as cisplatin and vincristine ranked even higher than high hematologic toxicity drugs such as doxorubicin and cyclophosphamide. This may be because drugs such as cisplatin and vincristine are more frequently used in chemotherapy regimens for children with WT and are often used in combination with other highly toxic chemotherapeutic drugs. Thus, the ranking of the feature importance of these variables may differ slightly from our general understanding of CIM risk factors. Nevertheless, the XGB model developed in this study still performed surprisingly well in predicting grade ≥ 2 CIM.

### 4.3. XGB Model Has Good Predictive Performance for Grade ≥ 2 CIM

Since the first mechanism model based on pharmacokinetics and pharmacodynamics was developed, other mathematical models for predicting CIM or investigating the relationship between a chemotherapy drug and changes in blood cell levels have been developed one after another. These mathematical models can simulate hematopoiesis, granulocytopoiesis, myelosuppression, and leukemia cytodynamics. Recently published reviews have provided a comprehensive overview and summary of various models [50,51], and studies have reported associations between the occurrence of CIM and genomic specificity [52,53,54]. Of these models, the maximum AUC of the model predicting FN or CIM occurrence is only 0.83. Notably, after evaluating the fitting effects of several models used in our study, we found that the XGB model had an AUC of up to 0.981 in the training set and 0.896 in the test set, with satisfactory sensitivity and specificity, as well as good stability. The calibration curve and DCA also suggested that the XGB model had good calibration and could promote clinical decision-making. In addition to good predictive performance, the XGB model we developed has other advantages: the modeling variables we selected were from the baseline data of hematological and biochemical tests before chemotherapy, and the information about the proposed chemotherapy regimen. These variables are readily available prior to drug administration. Children do not need to bear the expensive cost such as genomic marker detection, or the burden and pain caused by frequent laboratory tests.

### 4.4. Application of CIM Prediction Model in Clinical Practice

Translating clinical research results to clinical applications has been a significant challenge. The clinical decision support system (CDSS) helps doctors improve and enhance the efficiency of decision-making by providing systematic medical knowledge and in-depth analysis of medical records through a human–computer interaction model, thereby improving the quality of medical care [55]. CDSS is a vital bridge to facilitate the translation of clinical research into clinical application.

Considering the application scenarios of the CIM prediction model, we present the final model in the form of CDSS in our hospital HIS. Patients undergo hematological and biochemical tests after admission. The doctor then specifies the current chemotherapy regimen, followed by the system backstage immediately extracting the relevant data, calculating the CIM risk score through the model and outputting it via CDSS. Doctors can make appropriate treatment plans based on the predicted results. Despite the risk score module, the “Management Recommendations” module and the “Historical Trend” module that records the occurrence of CIM in previous chemotherapy cycles can greatly help doctors make better clinical decisions. To better demonstrate how our model works in reality and to further elaborate on the clinical applicability of the model, we ran the model in our hospital HIS to assess the risk of CIM in a particular child. Please refer to the Supplementary Materials (Figure S1) for sample cases and model results output interface.

By applying this approach, firstly, doctors can identify high-risk patients early and adopt appropriate management plans to improve patients’ prognosis. Secondly, the model calculations and results output are carried out automatically by the system backstage, eliminating the inconvenience of other predictive modeling tools requiring manual data input for the corresponding variables. Thirdly, the relevant data of CIM occurrence in each admission will be automatically stored in the system, which will be helpful for other related clinical studies in the future. All of the above fully reflect the practicability, accessibility, and high predictive efficiency of our model in clinical application.

### 4.5. Limitations and Prospects

However, our study also has some limitations. Firstly, the nature of the retrospective study may inevitably introduce some selection bias; secondly, the risk factors related to CIM, such as prealbumin, BMI, bile acid, bilirubin, etc., which have been reported in other studies [40,56], were not included in the model due to a large amount of missing data. This may be because doctors or patients have insufficient awareness of CIM and do not conduct relevant tests. Thirdly, the dynamic changes in blood cells may be able to predict the specific time when CIM occurs and finding this time point will help doctors develop more accurate prevention strategies for CIM. However, these data were also missing in this study. In addition, our sample size needs to be expanded to make more accurate predictions for different grades of CIM. Furthermore, our model has been successfully piloted in HIS with CDSS, and more data needs to be collected prospectively to further verify the model’s accuracy. Finally, different types of tumors may affect the occurrence of CIM, but only children with WT were included in this study. Therefore, the models that can be extended to other pediatric malignant solid tumors need further development. To summarize, a prospective clinical study with large samples and regularly collected data needs to be carried out. We are currently conducting animal experiments related to CIM in order to accurately predict the CIM by finding other more readily available indicators. We intend to validate these indicators in prospective clinical studies and incorporate them into the model for continuous calibration and optimization. Despite these limitations, to our knowledge, this study is the first to use ML algorithms to establish a predictive model for CIM in children with WT, achieving better predictive effects than other pharmacokinetic or mathematical models. Based on the construction method and clinical application approach of this ML model, a CIM prediction model that can be extended to other pediatric malignancies and facilitates widespread clinical applications can be expected.

## 5. Conclusions

The incidence of grade ≥ 2 CIM was not low in children with WT, which needs more attention. This study developed an ML-based prediction model to predict the risk of grade ≥ 2 CIM in WT children for the first time. The model has good predictive performance and stability and is also convenient for clinical application, which will help doctors identify patients at high risk of CIM earlier, and develop and implement individualized preventive medication strategies, thus reducing the disease burden and economic burden of CIM in children with WT. Based on this modeling and application approach, the extension of CIM prediction models to other pediatric malignancies is expected.

## Figures and Tables

**Table 1:** Comparison of baseline characteristics of patients in different data sets.

Variable	ALL (N = 1433)	Training Set (N = 1003)	Test Set (N = 430)	Statistic (*Z/χ*^2^)	*p* Value
Age (days), M (Q1–Q3)	1388 (807–2221)	1391 (810–2245)	1376 (803–2163)	−0.814	0.416
Sex				2.707	0.100
Female	674 (47.0%)	486 (48.5%)	188 (43.7%)		
Male	759 (53.0%)	517 (51.5%)	242 (56.3%)		
Weight (kg), M (Q1–Q3)	14.5 (11.5–19.0)	14.5 (11.5–19.0)	14.0 (11.5–18.0)	−0.844	0.398
BSA (m^2^), M (Q1–Q3)	0.61 (0.50–0.77)	0.61 (0.50–0.77)	0.59 (0.50–0.73)	−0.823	0.410
Tumor stage				6.561	0.161
I	116 (8.1%)	84 (8.4%)	32 (7.4%)		
II	224 (15.6%)	149 (14.9%)	75 (17.4%)		
III	495 (34.5%)	360 (35.9%)	135 (31.4%)		
IV	516 (36.0%)	360 (35.9%)	156 (36.3%)		
V	82 (5.7%)	50 (5.0%)	32 (7.4%)		
Risk classification (COG)				0.045	0.831
FH	1022 (71.3%)	717 (71.5%)	305 (70.9%)		
uFH	411 (28.7%)	286 (28.5%)	125 (29.1%)		
Chemotherapy cycles, M (Q1–Q3)	4.0 (2.0–9.0)	4.0 (2.0–8.0)	5.0 (2.0–9.0)	1.408	0.159
Hematologic index, M (Q1–Q3)					
Neutrophil percentage	0.59 (0.48–0.70)	0.59 (0.48–0.70)	0.58 (0.47–0.69)	−1.087	0.277
ANC (×10^9^/L)	3.50 (2.36–4.91)	3.53 (2.35–4.99)	3.49 (2.39–4.83)	−0.031	0.975
Monocyte percentage	0.04 (0.03–0.07)	0.04 (0.03–0.06)	0.04 (0.03–0.07)	−0.659	0.510
AMC (×10^9^/L)	0.28 (0.19–0.39)	0.28 (0.19–0.38)	0.30 (0.19–0.41)	−1.212	0.225
P–LCR (%)	24.2 (19.0–29.8)	24.2 (19.0–29.9)	24.4 (18.9–29.7)	−0.381	0.703
MCV (fL)	82.9 (78.7–87.6)	83.0 (78.6–87.6)	82.9 (78.8–87.5)	−0.283	0.777
MCHC (g/L)	325.0 (315.0–333.0)	325.0 (316.0–333.0)	325.0(315.0–333.0)	−0.100	0.920
MCH (pg)	27.1 (25.4–28.8)	27.0 (25.4–28.9)	27.1 (25.3–28.7)	−0.423	0.673
Lymphocyte percentage (%)	0.30 (0.20–0.43)	0.30 (0.20–0.43)	0.32 (0.21–0.43)	−0.842	0.400
ALC (×10^9^/L)	1.75 (0.95–3.11)	1.71 (0.97–2.99)	1.94 (0.93–3.36)	−1.220	0.223
WBC (×10^9^/L)	6.10 (4.32–8.73)	6.01 (4.27–8.73)	6.28 (4.45–8.74)	−0.809	0.419
RBC (×10^9^/L)	3.96 (3.52–4.36)	3.94 (3.51–4.33)	4.02 (3.53–4.41)	−1.427	0.154
RDW (%)	15.5 (14.0–17.3)	15.5 (14.0–17.3)	15.5 (14.1–17.5)	−0.551	0.582
ARD (fL)	47.0 (41.0–52.0)	47.0 (42.0–52.0)	47.0 (41.0–52.0)	−0.248	0.804
Hematocrit (%)	32.9 (29.9–35.5)	32.8 (29.9–35.3)	33.2 (29.8–35.9)	−1.257	0.209
PDW (fL)	11.0 (9.8–12.4)	11.0 (9.8–12.4)	11.1 (9.8–12.3)	−0.329	0.742
Thrombocytocrit (%)	0.31 (0.24–0.38)	0.31 (0.24–0.38)	0.32 (0.25–0.38)	−0.114	0.909
MPV (fL)	10.0 (9.3–10.7)	9.9 (9.3–10.7)	10.0 (9.3–10.7)	−0.274	0.784
PLT (×10^9^/L)	297.0 (227.0–387.0)	295.0 (223.0–390.0)	304.0 (238.0–378.0)	−0.903	0.366
Hgb (g/L)	107.0 (95.0–116.0)	107.0 (95.0–116.0)	107.0 (96.0–118.0)	−1.100	0.271
SII	575.2 (334.7–951.1)	579.8 (336.0–967.5)	569.4 (333.4–917.1)	−0.398	0.691
NLR	1.97 (1.12–3.37)	2.00 (1.13–3.44)	1.85 (1.09–3.24)	−0.996	0.319
PLR	162.8 (101.4–274.4)	169.7 (102.5–276.5)	149.6 (96.8–268.8)	−1.180	0.238
Urinalysis index					
pH	6.52 (6.00–7.00)	6.52 (6.00–7.00)	6.52 (6.00–7.00)	−0.433	0.665
Biochemical index					
LDH (U/L)	286.8 (227.0–418.4)	286.5 (228.0–418.4)	287.0 (225.0–418.4)	−0.246	0.806
UA (μmol/L)	284.8 (242.0–325.0)	284.8 (237.0–325.0)	284.8 (249.5–325.0)	−1.598	0.110
TBIL (μmol/L)	6.80 (4.00–8.10)	6.80 (4.00–8.20)	6.50 (4.00–7.80)	−0.970	0.332
TP (g/L)	63.8 (60.7–67.4)	63.8 (60.9–67.3)	63.8 (60.2–67.8)	−0.313	0.755
Globulin (g/L)	22.2 (19.3–24.4)	22.2 (19.1–24.4)	22.2 (19.6–24.2)	−0.202	0.840
Albumin (g/L)	41.7 (39.6–44.8)	41.7 (39.7–44.9)	41.7 (39.3–44.7)	−0.592	0.554
ALP (U/L)	175.8(133.1–197.3)	175.8 (134.0–199.0)	175.8(132.0–193.5)	−0.637	0.524
Scr (μmol/L)	34.3 (28.0–38.0)	34.3 (28.0–38.0)	34.3 (28.0–38.5)	−1.139	0.255
ALT (U/L)	21.7 (14.4–26.0)	21.1 (14.3–25.6)	22.5 (14.6–27.1)	−1.303	0.192
AST (U/L)	35.9 (28.1–40.0)	35.4 (28.0–39.4)	37.2 (29.0–41.3)	−2.270	0.023
Grade ≥ 2 CIM				0.001	0.977
With	594 (41.5%)	416 (41.5%)	178 (41.4%)		
Without	839 (58.5%)	587 (58.5%)	252 (58.6%)		

AMC: absolute monocyte count; P–LCR: platelet–large cell ratio; MCV: mean corpuscular volume; MCHC: mean corpuscular hemoglobin concentration; MCH: mean corpuscular hemoglobin; ALC: absolute lymphocyte count; WBC: white blood cell count; RBC: red blood cell count; RDW: red blood cell distribution width; ARD: absolute value of RBC distribution; PDW: platelet distribution width; MPV: mean platelet volume; PLT: platelet count; Hgb: hemoglobin; LDH: lactate dehydrogenase; UA: uric acid; TBIL: total bilirubin; TP: total protein; ALP: alkaline phosphatase; Scr: serum creatinine; ALT: alanine transaminase; AST: aspartate transaminase.

**Table 2:** Comparison of baseline characteristics of patients with and without CIM.

Variable	Grade ≥ 2 CIM		Statistic	*p* Value
	Without (N = 594)	With (N = 839)		
Age (days), M (Q1–Q3)	1554 (905–2478)	1294 (726–2022)	−4.432	<0.001
Sex			11.368	0.001
Female	248 (41.7%)	426 (50.8%)		
Male	346 (58.3%)	413 (49.2%)		
Weight (kg), M (Q1–Q3)	16.0 (12.0–20.0)	14.0 (11.0–18.0)	−5.388	<0.001
BSA (m^2^), M (Q1–Q3)	0.66 (0.52–0.80)	0.59 (0.49–0.73)	−5.385	<0.001
Tumor stage			1.915	0.751
I	51 (8.6%)	65 (7.8%)		
II	92 (15.5%)	132 (15.7%)		
III	211 (35.5%)	284 (33.9%)		
IV	211 (35.5%)	305 (36.4%)		
V	29 (4.9%)	53 (6.3%)		
Risk classification (COG)			3.011	0.083
FH	409 (68.9%)	613 (73.1%)		
uFH	185 (31.1%)	226 (26.9%)		
Chemotherapy cycles	5 (2.0–10.0)	4 (1.0–8.0)	5.574	<0.001
Hematologic index, M (Q1–Q3)				
Neutrophil percentage (%)	0.59 (0.48–0.71)	0.59 (0.47–0.69)	−1.398	0.162
ANC (×10^9^/L)	3.68 (2.65–4.74)	3.38 (2.06–5.10)	−2.631	0.009
Monocyte percentage (%)	0.04 (0.03–0.06)	0.05 (0.03–0.07)	−4.919	<0.001
AMC (×10^9^/L)	0.27 (0.19–0.37)	0.29 (0.20–0.40)	−2.650	0.008
P–LCR (%)	25.2 (20.1–31.3)	23.7 (18.3–28.4)	−3.726	<0.001
MCV (fL)	83.1 (79.4–87.3)	82.9 (78.1–87.7)	−0.853	0.393
MCHC (g/L)	328.0 (319.0–334.0)	322.0 (312.0–332.0)	−7.379	<0.001
MCH (pg)	27.3 (26.0–28.8)	26.9 (24.8–28.8)	−3.545	<0.001
Lymphocyte percentage (%)	0.31 (0.19–0.43)	0.30 (0.21–0.43)	−0.562	0.574
ALC(×10^9^/L)	1.85 (1.01–3.01)	1.70 (0.92–3.21)	−1.272	0.203
WBC (×10^9^/L)	6.35 (4.80–8.23)	5.98 (3.78–9.05)	−2.253	0.024
RBC (×10^9^/L)	4.21 (3.88–4.54)	3.73 (3.29–4.18)	−13.946	<0.001
RDW (%)	14.8 (13.7–16.1)	16.1 (14.4–18.3)	−9.918	<0.001
ARD (fL)	45.0 (41.0–49.0)	47.7 (42.0–54.0)	−6.889	<0.001
Hematocrit (%)	34.9 (33.0–36.8)	30.8 (27.9–33.6)	−18.756	<0.001
PDW (fL)	11.2 (10.0–12.6)	10.8 (9.7–12.1)	−4.376	<0.001
Thrombocytocrit (%)	0.29 (0.24–0.37)	0.34 (0.25–0.40)	−5.935	<0.001
MPV (fL)	10.0 (9.4–10.9)	9.9 (9.2–10.5)	−4.009	<0.001
PLT (×10^9^/L)	278.0 (218.0–345.0)	316.0 (237.0–413.0)	−5.989	<0.001
Hgb (g/L)	114.0 (107.0–121.0)	98.0 (89.0–110.0)	−19.054	<0.001
SII	516.2 (322.5–909.0)	616.0 (346.0–977.5)	−2.349	0.019
NLR	1.90 (1.13–3.65)	2.00 (1.11–3.22)	−0.650	0.516
PLR	140.0 (94.9–243.2)	180.1 (109.0–301.0)	−4.873	<0.001
Urinalysis index				
pH	6.52 (6.00–7.00)	6.52 (6.00–7.00)	−0.535	0.593
Biochemical index				
LDH (U/L)	275.0 (227.8–418.4)	297.1 (226.8–418.4)	−2.666	0.008
UA (μmol/L)	284.8 (242.0–305.1)	284.8 (241.0–335.0)	−3.076	0.002
TBIL (μmol/L)	6.86 (4.20–8.30)	6.10 (3.80–8.00)	−2.992	0.003
TP (g/L)	63.8 (62.0–68.1)	63.8 (59.9–67.0)	−4.537	<0.001
Globulin (g/L)	22.2 (19.2–23.9)	22.2 (19.5–24.8)	−1.555	0.120
Albumin (g/L)	42.5 (41.6–45.5)	41.7 (38.1–44.1)	−7.927	<0.001
ALP (U/L)	175.8 (160.0–204.8)	159.5 (118.9–188.0)	−8.870	<0.001
Scr (μmol/L)	34.3 (28.0–37.0)	34.3 (27.5–39.0)	−0.525	0.600
ALT(U/L)	21.0 (14.4–24.0)	22.0 (14.2–27.6)	−1.216	0.224
AST (U/L)	35.9 (28.8–38.1)	35.9 (28.0–42.0)	−1.416	0.157

AMC: absolute monocyte count; P–LCR: platelet–large cell ratio; MCV: mean corpuscular volume; MCHC: mean corpuscular hemoglobin concentration; MCH: mean corpuscular hemoglobin; ALC: absolute lymphocyte count; WBC: white blood cell count; RBC: red blood cell count; RDW: red blood cell distribution width; ARD: absolute value of RBC distribution; PDW: platelet distribution width; MPV: mean platelet volume; PLT: platelet count; Hgb: hemoglobin; LDH: lactate dehydrogenase; UA: uric acid; TBIL: total bilirubin; TP: total protein; ALP: alkaline phosphatase; Scr: serum creatinine; ALT: alanine transaminase; AST: aspartate transaminase.

**Table 3:** 40 clinical characteristic variables to be screened.

Variable	Missing Sample	Miss Rate (%)
Age	0	0.00
Sex	0	0.00
Weight	49	3.42
BSA	49	3.42
Tumor stage	1	0.07
Risk classification (COG)	48	3.35
Chemotherapy cycle	18	1.26
Neutrophil percentage	5	0.35
ANC	18	1.26
Monocyte percentage	8	0.56
AMC	45	3.14
P–LCR	94	6.56
MCV	3	0.21
MCHC	3	0.21
MCH	2	0.14
Lymphocyte percentage	6	0.42
ALC	16	1.12
WBC	2	0.14
RBC	1	0.07
RDW	4	0.28
ARD	102	7.12
Hematocrit	3	0.21
PDW	86	6.00
Thrombocytocrit	114	7.96
MPV	81	5.65
PLT	2	0.14
Hgb	1	0.07
SII	16	1.12
NLR	16	1.12
PLR	16	1.12
LDH	219	15.28
UA	204	14.24
TBIL	220	15.35
TP	219	15.28
Globulin	220	15.35
Albumin	219	15.28
ALP	220	15.35
Scr	205	14.31
ALT	221	15.42
AST	220	15.35

AMC: absolute monocyte count; P–LCR: platelet–large cell ratio; MCV: mean corpuscular volume; MCHC: mean corpuscular hemoglobin concentration; MCH: mean corpuscular hemoglobin; ALC: absolute lymphocyte count; WBC: white blood cell count; RBC: red blood cell count; RDW: red blood cell distribution width; ARD: absolute value of RBC distribution; PDW: platelet distribution width; MPV: mean platelet volume; PLT: platelet count; Hgb: hemoglobin; LDH: lactate dehydrogenase; UA: uric acid; TBIL: total bilirubin; TP: total protein; ALP: alkaline phosphatase; Scr: serum creatinine; ALT: alanine transaminase; AST: aspartate transaminase.

**Table 4:** Frequency of use of each chemotherapy drug.

Drug	Relative Frequency	Frequency
Bleomycin	0.001	1
Fluorouracil	0.006	9
Topotecan	0.011	16
Vindesine	0.012	17
Ifosfamide	0.017	23
Cisplatin	0.124	172
Doxorubicin	0.126	176
Epirubicin	0.175	243
Carboplatin	0.254	354
Etoposide	0.342	476
Actinomycin D	0.348	485
Cyclophosphamide	0.504	701
Vincristine	0.703	978

**Table 5:** Variables finally included in the model.

Variable (n = 19)	IV
Hgb	1.770
RBC	0.708
ALP	0.422
RDW	0.392
WBC	0.372
ANC	0.369
Albumin	0.328
MCHC	0.243
PLT	0.213
Chemotherapy cycles	0.082
Coadministration of highly toxic chemotherapy drug	0.061
Cisplatin	0.028
Vincristine	0.022
Epirubicin	0.013
Carboplatin	0.007
Actinomycin D	0.005
Etoposide	0.001
Cyclophosphamide	0.000
Doxorubicin	0.000

IV: information value; Hgb: hemoglobin; RBC: red blood cell count; ALP: alkaline phosphatase; RDW: red blood cell distribution width; WBC: white blood cell count; MCHC: mean corpuscular hemoglobin concentration; PLT: platelet count.

**Table 6:** Evaluation of fitting effect of each model.

Model	AUC		PSI
	Training Set	Test Set	
XGB	0.981	0.896	0.033
CatBoost	0.996	0.888	0.086
RF	0.842	0.856	0.015
SVM	0.930	0.849	0.066
LR	0.843	0.842	0.007
LASSO	0.843	0.842	0.007

XGB: extreme gradient boosting; LR: logistic regression; RF: random forest; LASSO: least absolute shrinkage and selection operator; SVM: support vector machine; PSI: population stability index.

**Table 7:** Evaluation of authenticity of each model.

Model	Best Cutoff	TPR	TNR	ACC	PPV
XGB	0.529	76.2%	93.3%	83.3%	94.1%
RF	0.569	68.3%	88.2%	76.5%	89.1%
CatBoost	0.585	75.0%	90.4%	81.4%	91.7%
SVM	0.581	75.0%	84.3%	78.8%	87.1%
LR	0.687	66.3%	88.8%	75.6%	89.3%
LASSO	0.685	66.3%	88.8%	75.6%	89.3%

TPR: sensitivity; TNR: specificity; ACC: precision; PPV: precision; XGB: extreme gradient boosting; LR: logistic regression; RF: random forest; LASSO: least absolute shrinkage and selection operator; SVM: support vector machine.

## Data Availability

The datasets used and/or analyzed during the current study are available from the corresponding author upon reasonable request.

## Supplementary Materials

The following supporting information can be downloaded at: https://www.mdpi.com/article/10.3390/cancers15041078/s1. Figure S1. Case example: the interface of CIM prediction model output results. The text in the figure has been translated into English.
