# Supplementary material for: Development of a Machine Learning-Based Prediction Model for Chemotherapy-Induced Myelosuppression in Children with Wilms’ Tumor
Source: Cancers (Basel). 2023 Feb 8;15(4):1078. doi: 10.3390/cancers15041078 (PMC9954251; doi:10.3390/cancers15041078)
Supplement: Supplementary file 1 [file cancers-15-01078-s001.zip › cancers-2189164-SI.pdf]

Supplementary Figure and Descriptions

To better demonstrate how our model works in reality and to further elaborate on the clinical applicability of the model so that readers have a better understanding of the model's results, we ran the model in our hospital HIS to assess the risk of CIM in a particular child (Figure S1).

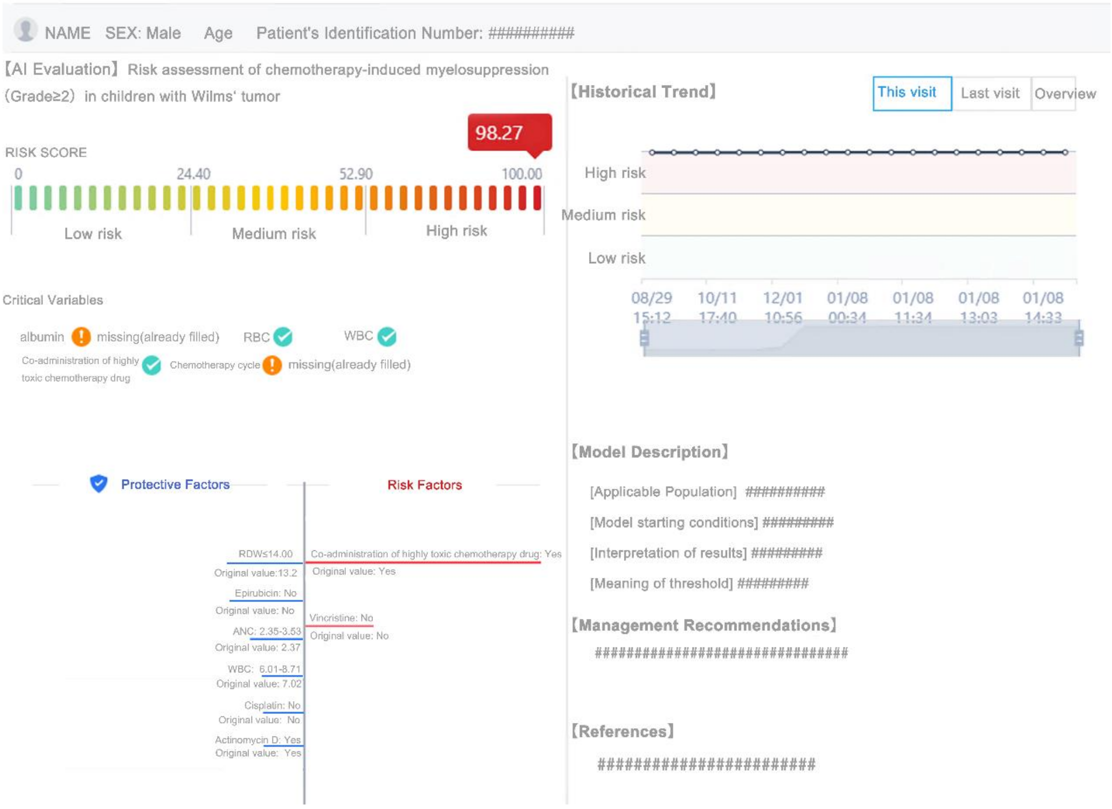

Figure S1. Case example: the interface of CIM prediction model output results. The text in the figure has been translated into English.

A detailed explanation of the figure and the illustration of how the model works

Herein, I will illustrate this figure in detail with the specific clinical data of this case.

The model is used as follows: the patient receives some hematological examinations immediately after admission to the hospital and a chemotherapy regimen is developed by the doctor according to the child's condition. After the results of these examinations are available, the model will be launched in HIS, and the model will automatically collect the results of relevant examination indexes and chemotherapy drugs, and immediately output the risk assessment score of CIM after a quick calculation in the back-stage.

This patient has been diagnosed with WT and this admission is proposed for the 1st chemotherapy cycle. His indicators collected by the model are as follows: Hgb *117g/L*, WBC *7.02×10⁹/L*, ALP *210 U/L*, Co-administration of highly toxic chemotherapy drug *YES*, Albumin *38.5 g/L*, RBC *4.3 ×10¹²/L*, ANC *2.37 ×10⁹/L*, MCHC *337 g/L*, PLT *277×10⁹/L*, RDW *13.2%*, Vincristine *NO*, Chemotherapy cycle *1*, Epirubicin *NO*, Cisplatin *NO*, Doxorubicin *NO*, Cyclophosphamide *NO*, Carboplatin *NO*, Etoposide *NO*, Actinomycin D *YES*.

Note that in this module of the AI Evaluation, in addition to the risk score, some critical variables in this calculation are also displayed, including albumin, RBC, WBC, chemotherapy cycle, and Co-administration of highly toxic chemotherapy drugs. Among these variables, the model failed to automatically extract data for albumin and chemotherapy cycles. Fortunately, our model allows for the manual imputation of important missing data. This work is often necessary because more complete data may yield more accurate predictions.

As shown in the figure, the patient has a CIM risk score of 98.27, which is considered high risk. So which variables are risk factors and which are protective factors for this patient?

As we mentioned before, different from the traditional logistics regression algorithm, in the operation process of the machine learning prediction model, the protective factors and risk factors are often different for different samples or individuals. For this, our model gives a personalized interpretation. As shown in the figure, for this patient, the reference values above each blue or red line can be interpreted as a distinction between protective and risk factors. For example, since this patient has an RDW≤14.00, RDW is a protective factor for him, and vice versa. In this way, for this patient, we can better understand the contribution of each variable to the risk score. At the same time, we also found from the figure that " Vincristine *NO*" is actually a risk factor, which may be explained as follows. For most children with WT, vincristine is a commonly used chemotherapy drug, often alone or in combination with actinomycin D, for the treatment of low-stage WT. However, for some patients with high tumor stage, carboplatin, etoposide, cyclophosphamide and other such chemotherapy drugs with high hematotoxicity may be chosen with an increasing risk of CIM. We suspect that such a pattern may have been identified in the modeling process, so " Vincristine *NO*" became a risk factor for this child.

The modules of the historical trend, model descriptions and management recommendations in the figure have been described in the original manuscript and will not be repeated here.
